# DGFI-Net: dual-branch guided feature interaction network for brain tumor segmentation

**DOI:** 10.3389/fphys.2026.1817401

**Published:** 2026-04-27

**Authors:** Longyun Zhao, Xiaoliang Jiang, Qile Zhang

**Affiliations:** 1 Special Equipment Institute, Hangzhou Polytechnic University, Hangzhou, China; 2 Faculty of Electrical Engineering and Computer Science, Ningbo University, Ningbo, China; 3 College of Mechanical Engineering, Quzhou University, Quzhou, China; 4 Department of Rehabilitation, The Quzhou Affiliated Hospital of Wenzhou Medical University, Quzhou People’s Hospital, Quzhou, China

**Keywords:** attention-guided feature refinement block, brain tumor segmentation, dual-branch architecture, efficient context refinement block, lightweight reinforcement module

## Abstract

**Introduction:**

Accurate brain tumor segmentation is essential for clinical diagnosis and treatment planning. However, the complex anatomical structures, blurred tumor boundaries, and large inter-patient variability pose significant challenges to existing segmentation models. To address these issues, we propose DGFI-Net, a dual-branch guided feature interaction network.

**Methods:**

DGFI-Net introduces an auxiliary branch to guide the main branch’s feature learning via hierarchical interaction. Specifically, the main encoding branch is equipped with an efficient context refinement block (ECRB) to capture long-range contextual dependencies. In parallel, the auxiliary encoding branch employs an attention-guided feature refinement block (AGFRB) to emphasize salient tumor regions. At the bottleneck stage of the main encoding branch, we further introduce a lightweight reinforcement module (LRM) to strengthen high-level semantic representations. During the decoding process, the auxiliary branch continuously guides the main branch by transferring both encoding and decoding feature.

**Results and Discussion:**

Extensive experiments on three public brain tumor datasets (BrainTumor1, BrainTumor2, and BrainTumor3) and a pituitary adenoma dataset collected from Quzhou People’s Hospital demonstrate the superiority of DGFI-Net. The proposed method achieves Dice scores of 0.8402, 0.9011, 0.8040, and 0.9117 on the four datasets, with corresponding Mcc values of 0.8387, 0.8984, 0.8021, and 0.9100, and Jaccard values of 0.7318, 0.8213, 0.6899, and 0.8388. Moreover, comprehensive ablation studies are conducted to verify the effectiveness of each proposed component. These results validate the superiority of the proposed guided dual-branch interaction paradigm for complex medical image segmentation tasks.

## 1 Introduction

Brain tumor segmentation constitutes a fundamental component in modern computer-aided diagnosis systems, and its accuracy directly affects clinical decision-making, treatment planning, and patient prognosis evaluation. In routine clinical practice, due to the diversity of tumor types, variations in imaging protocols, and differences in individual anatomical structures, brain tumor appearances often exhibit significant heterogeneity in terms of shape, size, location, and intensity distribution. Moreover, the boundaries between tumor tissues and surrounding normal regions are frequently ambiguous, especially in infiltrative or low-contrast areas, which further complicates precise delineation. These characteristics not only hinder the visual interpretation of medical images but also increase the risk of inaccurate diagnosis and suboptimal therapeutic decisions. Therefore, achieving efficient identification and precise localization of brain tumor regions has become a crucial task for improving the reliability and intelligence level of modern clinical diagnosis systems.

In recent years, deep learning-based segmentation methods, particularly convolutional neural networks (CNNs) (Ajabani et al., 2025; Yang et al., 2026; Ge et al., 2026), have achieved remarkable success in automatically learning hierarchical feature representations from medical images, significantly reducing the reliance on handcrafted features and expert intervention. Benefiting from their strong nonlinear modeling capability and multi-level feature extraction mechanisms, a growing number of studies have been devoted to the investigation of brain tumor segmentation. Among them, Jadhav et al (Jadhav et al., 2026) proposed a dual SE-based architecture that performs channel-wise calibration across multiple semantic levels by adaptively reweighting feature responses at different network depths. Zhou et al (Zhou et al., 2025a) introduced a contrastive perception learning framework to improve the discriminative capability of feature representations by explicitly modeling the differences between similar and dissimilar samples. Shan et al (Shan et al., 2026) introduced a static task prompt module designed to strengthen the network’s target perception capability by explicitly embedding task-specific priors into the feature learning process. Jin et al (Jin et al., 2025) proposed a hierarchical channel-level attention decoder that effectively integrates multi-scale information by adaptively emphasizing informative feature channels at different semantic levels. Reddy et al (Reddy et al., 2025) introduced an axial slice attention mechanism that models contextual dependencies along different anatomical axes, enabling the network to emphasize informative slices and capture long-range structural correlations more effectively. Rani et al (Rani et al., 2025) employed a spatial-frequency-guided decoder to enhance feature representation by jointly exploiting spatial structures and frequency-domain cues. Fan et al (Fan et al., 2026) proposed a four-branch encoder structure, where each branch uses a different attention mechanism. This design allows the model to learn more discriminative features by focusing on informative regions while minimizing irrelevant information. He et al (He et al., 2026) combined multi-head cross-attention with state-space modeling to enhance feature representation and capture long-range dependencies. Ahmad et al (Ahmad et al., 2025) proposed a lightweight Inception-based U-Net architecture, which leverages Inception modules to capture multi-scale feature representations. He et al. (2025a) developed an active learning network that improves performance while reducing the need for large amounts of annotated data.

Despite these encouraging advances, existing approaches still face several limitations when dealing with the intrinsic complexity of brain tumors. Most conventional models adopt a single-branch architecture, which often struggles to simultaneously capture long-range contextual dependencies and preserve fine-grained boundary details. In addition, simple feature fusion strategies, such as direct concatenation or summation, fail to fully exploit the complementary information among multi-level representations. To overcome these limitations, we propose DGFI-Net, a dual-branch guided feature interaction network that explicitly models cross-branch information exchange to achieve more effective contextual modeling and boundary-aware refinement. The main contributions of this work can be summarized as follows:

1. We propose DGFI-Net, a novel dual-branch guided feature interaction network for brain tumor segmentation, in which an auxiliary branch is explicitly introduced to guide the feature learning of the main branch through hierarchical and progressive cross-branch interaction. This design enables effective information exchange between branches and facilitates complementary integration of contextual semantics and fine-grained structural details.
2. To enhance feature representation capability, we design two specialized refinement modules for the dual branches. Specifically, the main encoding branch is equipped with the efficient context refinement block to capture long-range contextual dependencies while preserving discriminative semantic information, whereas the auxiliary encoding branch incorporates the attention-guided feature refinement block to emphasize salient tumor regions and suppress irrelevant background responses via adaptive attention modulation.
3. At the bottleneck stage of the main encoding branch, we further introduce a lightweight reinforcement module to strengthen high-level semantic representations and improve global context awareness, while maintaining a low computational overhead. This module effectively enhances the robustness and expressiveness of deep features, thereby facilitating more accurate segmentation.

## 2 Related work

### 2.1 Dual-branch architecture

Dual-branch architectures offer a powerful mechanism for enhancing feature representation by enabling parallel feature extraction and explicit cross-branch interaction, which helps exploit complementary information from different semantic perspectives. Consequently, this architectural paradigm has gained increasing attention in recent years. For instance, Sun (Sun, 2025) proposed a dual-branch transformer-convolution framework with cross-branch fusion, where dynamic deformable convolution enhances local feature modeling in the CNN branch, and a mobile window-based attention module captures long-range dependencies in the transformer branch. He et al. (He et al., 2025b) developed a graph-based dual-branch framework, where one branch is dedicated to global relational reasoning to capture holistic contextual information, while the other branch employs local graph attention to model fine-grained structural details. Tang et al (Tang et al., 2025) designed a dual-path hybrid architecture that integrates CNNs and transformers, where the convolutional pathway emphasizes fine-grained local patterns, while the transformer pathway focuses on capturing long-range contextual relationships. Li et al (Li et al., 2025a) designed a two-stream framework that processes features at different resolutions, where one pathway preserves detailed spatial information while the other captures coarse-grained contextual semantics. Dong et al (Dong et al., 2026) developed a two-stream encoding architecture that separately models fine-grained local patterns and global contextual semantics and employed an adaptive gating mechanism to dynamically regulate and optimize cross-branch feature integration.

### 2.2 Attention mechanism

Attention mechanisms enhance feature representation by enabling networks to selectively emphasize informative patterns and suppress irrelevant responses. By dynamically weighting spatial or channel-wise features, these mechanisms facilitate more discriminative learning and thus have been widely explored in recent studies. For example, Xiao et al (Xiao et al., 2025) proposed a selective dual-attention fusion gate to adaptively integrate complementary features and enhance discriminative representation learning. Zhang et al (Zhang et al., 2025) introduced an attention-guided knowledge distillation module that facilitates the transfer of task-relevant representations from teacher models to student networks. Wu and Tang (Wu and Tang, 2026) proposed a cross-feature attention module to preserve discriminative information and mitigate representation degradation in deeper network layers. Fang et al (Fang et al., 2025) introduced a dual-stage frequency-spatial attention module to jointly model global contextual cues and fine-grained structural details for more precise localization. Ma et al (Ma et al., 2025) proposed a multi-scale channel attention module that leverages convolution kernels of different sizes to capture scale-aware feature representations. Zhou et al (Zhou et al., 2025b) introduced a fuzzy axial attention module that performs self-attention separately along the height and width dimensions to model long-range spatial dependencies more efficiently.

## 3 Methods

### 3.1 Network architecture of DGFI-Net

**Figure 1:**
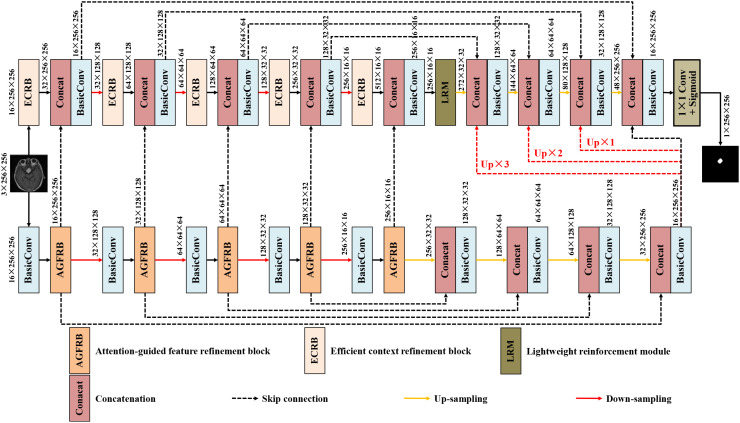
Network architecture of DGFI-Net.

Dual-branch architectures have been widely adopted due to their ability to learn complementary feature representations from multiple pathways. However, most existing frameworks rely on either serial or parallel designs, which limit the effective utilization of such complementary information. In serial configurations, the sequential propagation of features may lead to the gradual loss of fine-grained details, while in parallel structures, interactions between branches are often restricted to simple operations such as concatenation or summation, resulting in insufficient feature exchange. To overcome these limitations, we propose DGFI-Net, a dual-branch guided feature interaction network that facilitates deep and hierarchical information integration between two parallel U-shaped encoder-decoder branches, as illustrated in **Figure 1**. Specifically, DGFI-Net adopts a dual-branch architecture consisting of a main branch and an auxiliary branch. In the main encoding branch, we introduce the efficient context refinement block to capture long-range contextual dependencies while preserving discriminative semantic representations. Meanwhile, the auxiliary encoding branch is equipped with attention-guided feature refinement block, which selectively enhance tumor-relevant patterns and attenuate background interference via adaptive attention weighting. At the bottleneck stage of the main encoding branch, we further embed a lightweight reinforcement module to enhance the representational capacity of high-level semantic features while maintaining low computational overhead. To facilitate effective cross-branch interaction, features from the auxiliary branch are progressively transferred to the main branch during encoding, enabling hierarchical guided feature learning. During decoding, auxiliary features are downsampled at multiple scales (×1, ×2, and ×3) and fused with the corresponding features in the main decoder. This hierarchical fusion mechanism promotes effective cross-branch information flow, enhances structural consistency, and improves boundary-aware refinement. Finally, the aggregated features are refined and passed through a convolutional layer followed by a Sigmoid activation function to generate the final segmentation map. Compared with conventional dual-branch designs, DGFI-Net enables deep and progressive feature interaction, provides explicit cross-branch guidance, and effectively integrates global contextual information with fine-grained structural details.

### 3.2 Efficient context refinement block

**Figure 2:**
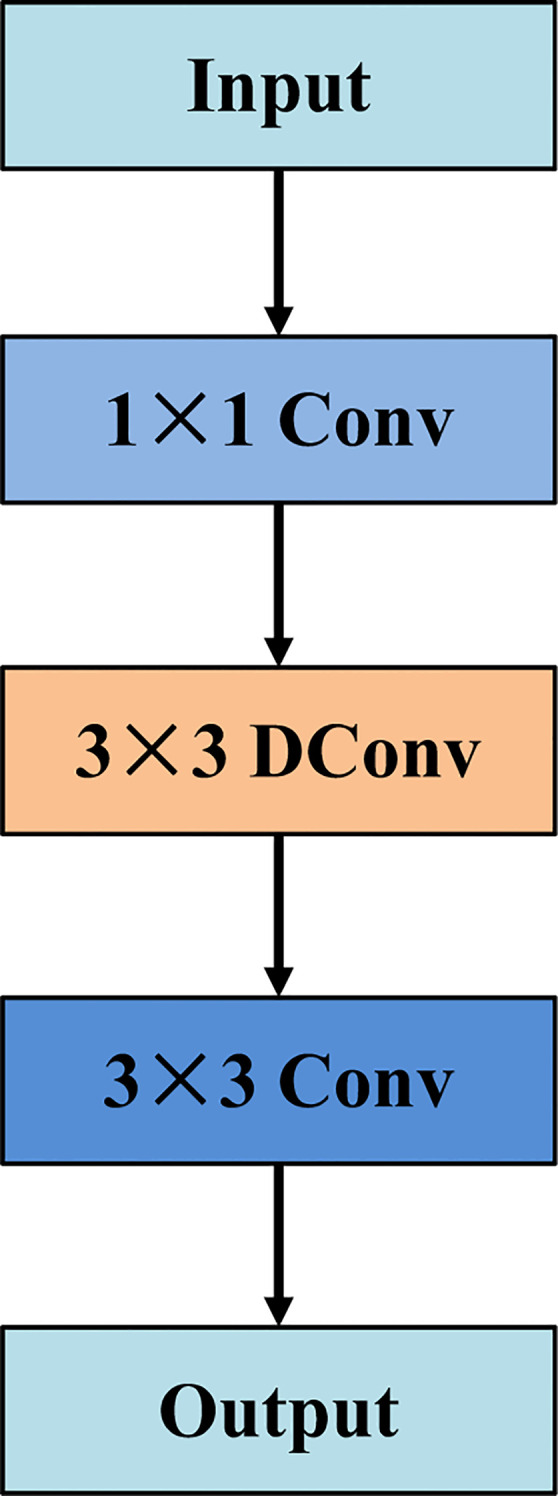
Structure of efficient context refinement block.

In conventional U-Net-based architectures, the encoder typically adopts a double convolution structure composed of two consecutive 3×3 convolutional layers. Although this configuration is well-suited for modeling local spatial patterns, it lacks the capacity to effectively capture long-range contextual dependencies. To overcome this limitation, we propose an efficient context refinement block, which is specifically embedded within the encoding stages of the main branch, as illustrated in **Figure 2**. Specifically, the ECRB begins with a 1×1 convolution to perform channel-wise transformation and dimensional regulation, enabling efficient cross-channel interaction while alleviating feature redundancy. Subsequently, a 3×3 dilated convolution (DConv) is introduced to enlarge the receptive field without increasing the parameter count or compromising spatial resolution. This operation allows the block to capture broader contextual dependencies, which are crucial for characterizing the global structure of tumors and their surrounding tissues. Finally, a 3×3 convolution is applied to further refine local spatial details and improve the discriminability of the extracted features. Compared with the conventional double convolution block, the ECRB effectively integrates global contextual modeling and local feature refinement within the main branch encoder. By enlarging the receptive field through dilated convolution while maintaining computational efficiency, it achieves a better balance between contextual awareness and detail preservation, thereby improving the representation capability of encoder features for complex medical image segmentation tasks.

### 3.3 Attention-guided feature refinement block

**Figure 3:**
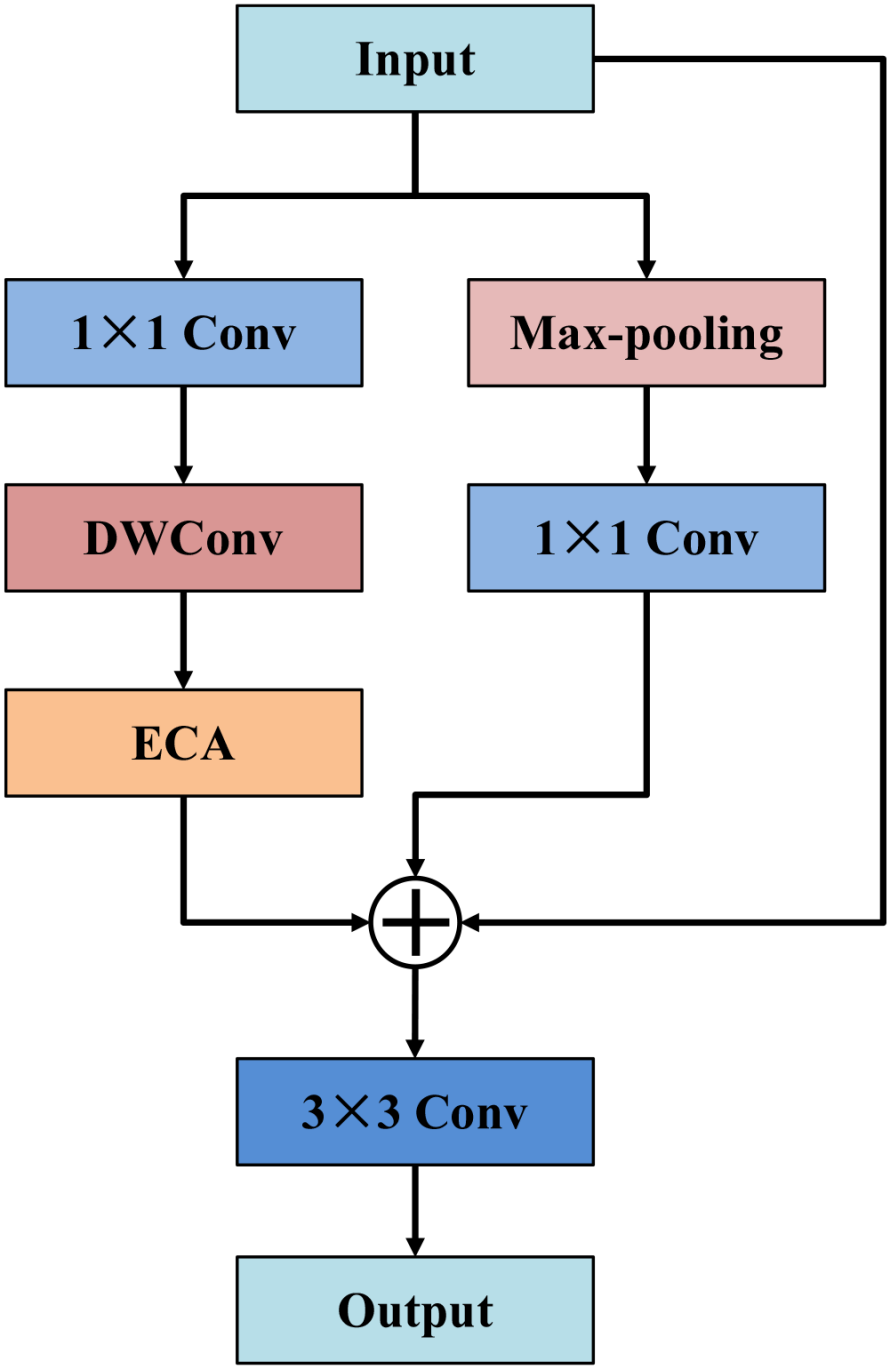
Structure of attention-guided feature refinement block.

As illustrated in **Figure 3**, the attention-guided feature refinement block is designed as the core encoding module in the auxiliary branch, aiming to enhance feature representation by explicitly emphasizing tumor-relevant patterns and suppressing background interference. Specifically, the input feature map is processed through two parallel pathways to extract complementary representations. In the upper branch, a max-pooling operation is first applied to highlight dominant activations and salient structural patterns, followed by a 1×1 convolution for channel-wise re-encoding and dimensional alignment. This pathway focuses on capturing prominent contextual responses in a compact manner. In the lower branch, a 1×1 convolution is first employed to reduce feature redundancy and enhance channel interactions, followed by a depthwise convolution (DWConv) to efficiently model local spatial details with low computational cost. To further improve feature selectivity, an efficient channel attention (ECA) module is incorporated to dynamically recalibrate channel responses, allowing the network to highlight tumor-relevant information and attenuate background interference. After dual-path processing, the outputs of both branches and the original input features are simultaneously integrated through an element-wise summation operation. This fusion strategy enables the network to jointly exploit salient contextual cues, attention-enhanced local details, and the original semantic information. Finally, the combined representation is passed through a 3×3 convolution to further refine spatial patterns and enhance the discriminative capability of the output features. Through this carefully designed structure, AGFRB effectively combines max-pooling-based saliency extraction, depthwise convolution-based local modeling, and attention-driven channel recalibration. Unlike the main branch, which focuses on contextual modeling, the auxiliary branch equipped with AGFRB is specifically designed to capture fine-grained discriminative cues and provide complementary guidance to the main branch, thereby enhancing cross-branch feature learning and overall segmentation performance.

**Figure 4:**
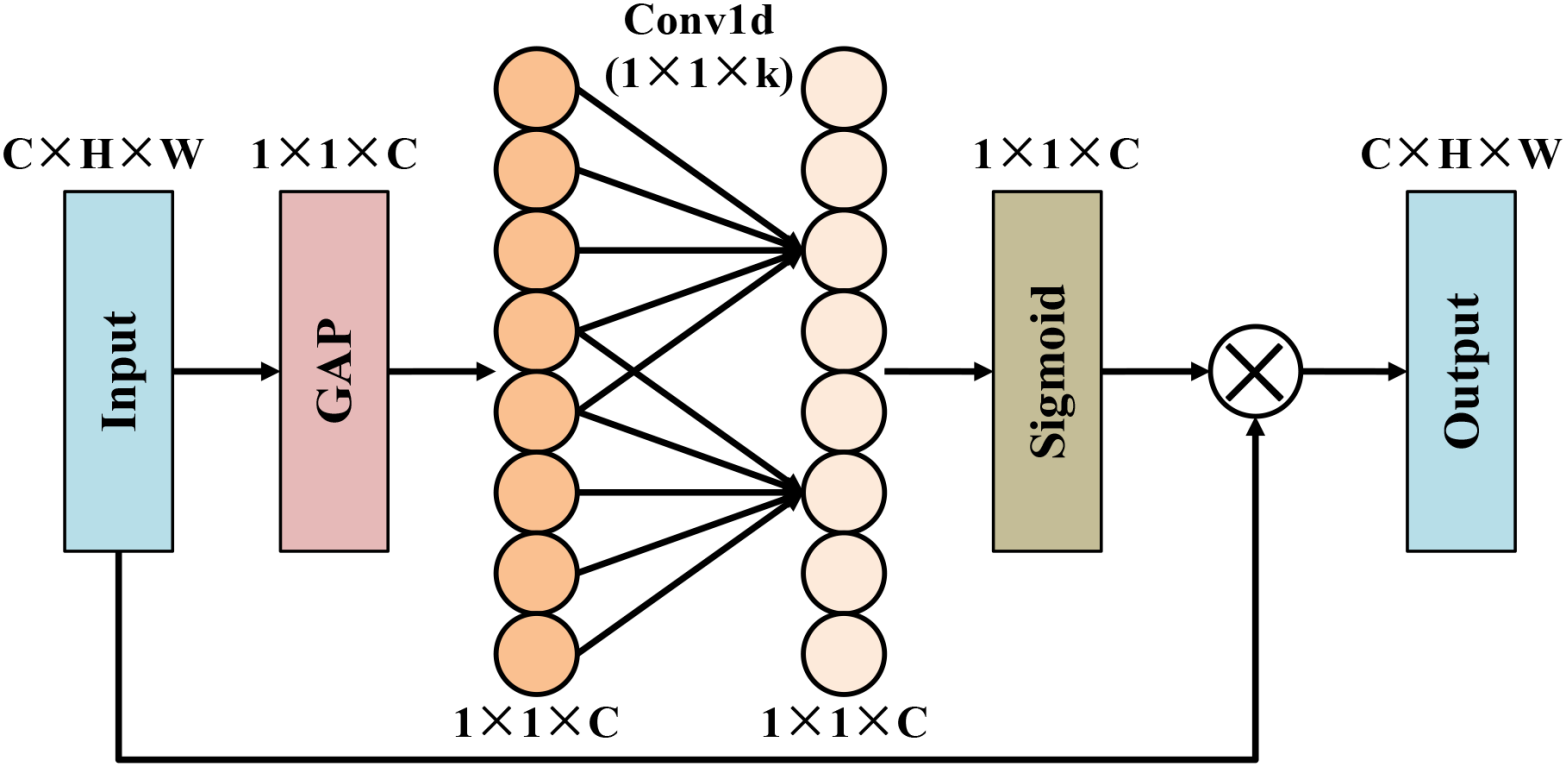
Structure of efficient channel attention module.

As illustrated in **Figure 4**, the efficient channel attention module (Li et al., 2025b) aims to enhance feature representation by adaptively recalibrating channel-wise responses with minimal computational overhead. Unlike conventional channel attention mechanisms that rely on fully connected layers, ECA models channel dependencies through a lightweight one-dimensional convolution, thereby avoiding dimensionality reduction and excessive parameters. Specifically, global average pooling is first applied to aggregate spatial information into a channel descriptor, which is then processed by a 1D convolution with an adaptive kernel size to capture local cross-channel interactions. The resulting attention weights are activated by a sigmoid function and used to reweight the input feature map via channel-wise multiplication. This design allows ECA to emphasize informative channels while suppressing irrelevant ones, achieving an effective balance between representational power and computational efficiency.

### 3.4 Lightweight reinforcement module

**Figure 5:**
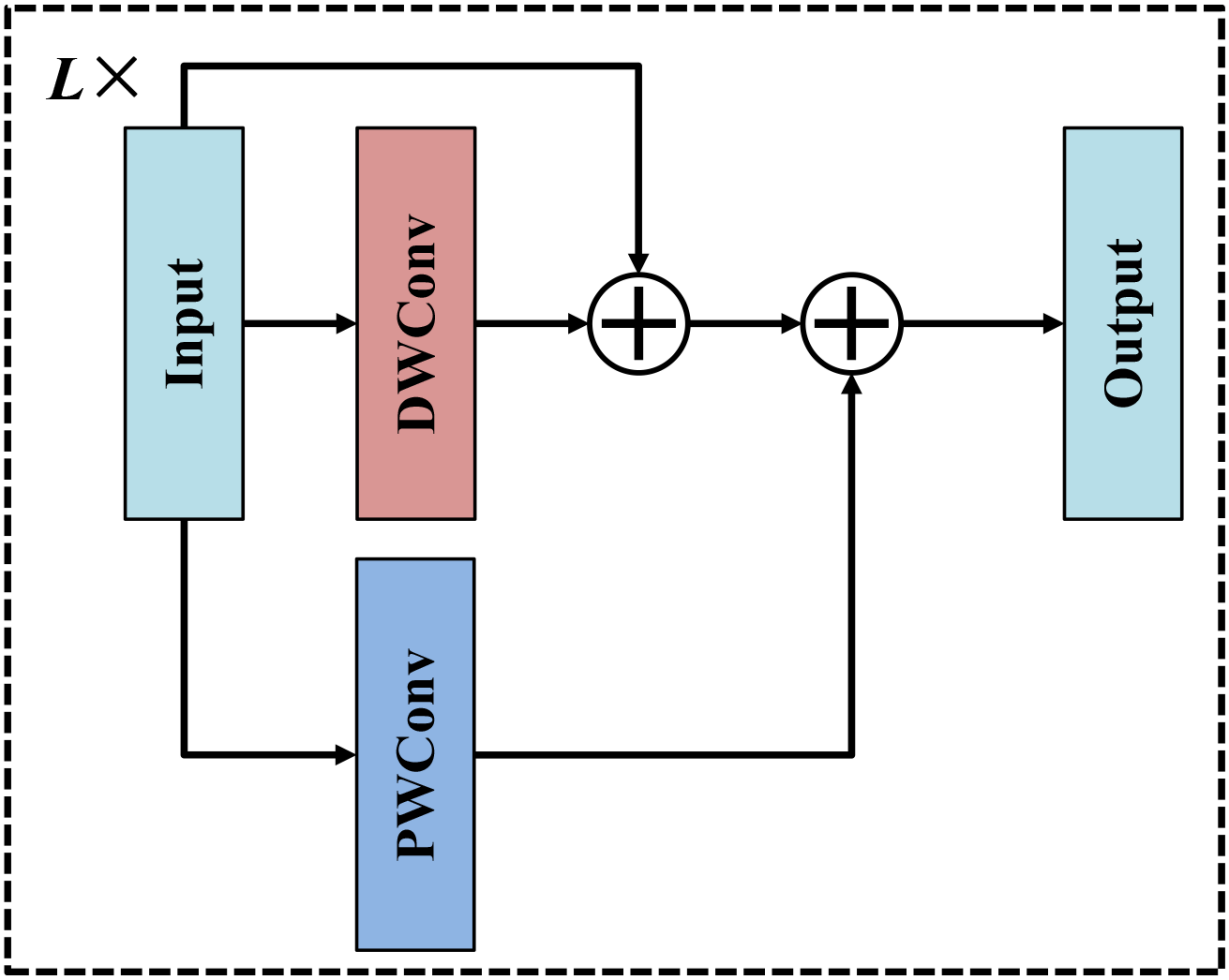
Structure of lightweight reinforcement module.

To strengthen high-level semantic representation and enhance global context perception while maintaining low computational complexity, we introduce a lightweight reinforcement module at the bottleneck stage of the main encoding branch, as illustrated in **Figure 5**. Specifically, the lightweight reinforcement module consists of L cascaded layers, each designed to jointly exploit spatial and channel-wise feature representations through the combination of DWConv and pointwise convolution (PWConv). In each layer, a DWConv with a k×k kernel is first applied to independently process each channel, enabling the capture of fine-grained spatial patterns and subtle local contextual cues. To preserve the original spatial information and stabilize feature transformation, a residual shortcut is introduced. In parallel, a PWConv with a 1×1 kernel is employed to model cross-channel interactions and facilitate efficient information mixing. The outputs from the DWConv branch, the PWConv branch, and the residual pathway are then aggregated via element-wise summation, enabling complementary integration of spatial details, channel-wise semantics, and original feature representations with negligible computational overhead. Through the progressive stacking of multiple such layers, the proposed LRM achieves a more effective balance between representational capacity and computational efficiency, thereby improving global context modeling without introducing significant parameter overhead, which is particularly beneficial for complex medical image segmentation tasks.

### 3.5 Loss function

In this work, we employ Dice loss (Luo et al., 2024; Yuan et al., 2024) as the optimization objective due to its effectiveness in handling class imbalance, which is prevalent in segmentation tasks with small foreground regions. Unlike pixel-wise losses, Dice loss directly measures region-level overlap, promoting better structural consistency and boundary accuracy. Its formulation is defined in Equation 1:

(1) $$L_{Dice}=1-\frac{2\sum_{i=1}^{N}y_{i}{\hat{y}}_{i}}{\sum_{i=1}^{N}y_{i}^{2}+\sum_{i=1}^{N}{\hat{y}}_{i}^{2}}$$

where is the number of pixels, and denote the labeled value and predicted value.

## 4 Experiments and discussion

### 4.1 Dataset

**Figure 6:**
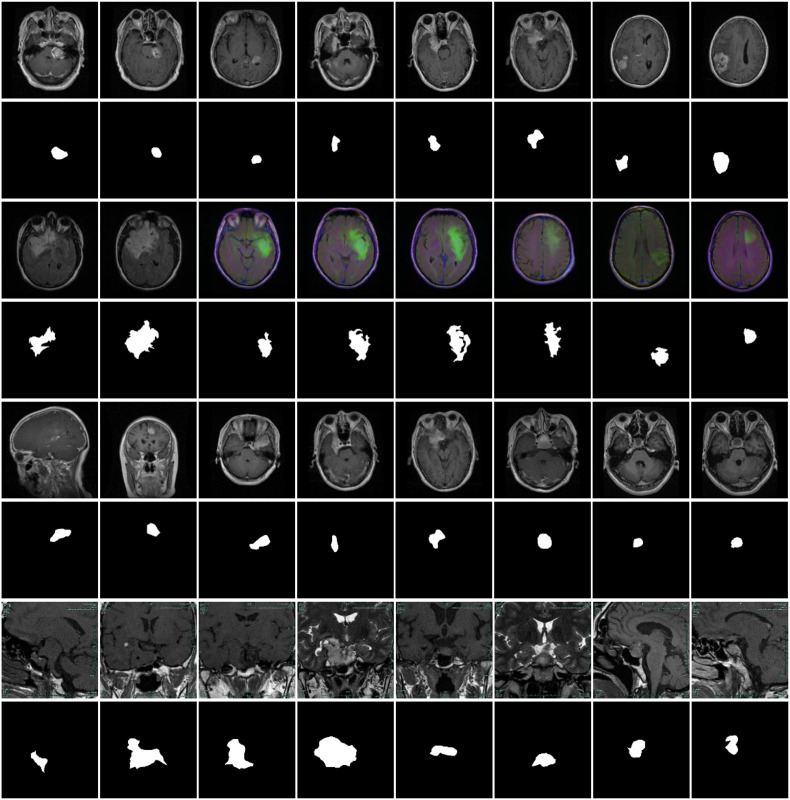
Representative examples from the four brain tumor datasets. The first two rows present the original images and their corresponding masks from the BrainTumor1 dataset. The third and fourth rows are the original images and annotated labels from BrainTumor2 dataset. The fifth and sixth rows show the original images and their corresponding annotations from BrainTumor3 dataset. The last two rows illustrate the original images and their corresponding annotations from pituitary adenoma dataset.

In our experiments, three openly accessible brain tumor segmentation benchmark datasets and a pituitary adenoma dataset were adopted to comprehensively assess the performance of the proposed DGFI-Net. The first dataset, referred to as BrainTumor1, contains 3,064 annotated MRI image pairs, which are partitioned into 1,839 samples for training, 612 for testing, and 613 for validation. This dataset includes a diverse range of tumor appearances and imaging conditions, providing a representative benchmark for evaluation. The second dataset, BrainTumor2, consists of 1,373 MRI images, among which 824 images are allocated for training, 274 for testing, and 275 for validation. The third dataset, BrainTumor3, includes a total of 1,426 MRI brain scans, which are divided into 856 training samples, 285 validation samples, and 285 test samples. The fourth dataset, pituitary adenoma dataset, was collected from Quzhou People’s Hospital and annotated by clinical experts. It contains 2,105 MRI images of pituitary adenomas, which are split into 1,400 training samples, 305 validation samples, and 400 test samples for experimental evaluation. Representative examples from these four datasets are illustrated in **Figure 6**.

### 4.2 Evaluation metrics

To comprehensively evaluate the performance of DGFI-Net, three commonly adopted metrics were utilized, including Dice (Wan et al., 2025a; Li et al., 2022), Matthews correlation coefficient (Mcc) (Wan et al., 2025b; Shang et al., 2024), and Jaccard (Yang et al., 2024; Yeung et al., 2023). The Dice, Mcc, and Jaccard metrics are defined in Equations 2, 3 and 4.

(2) $$Dice=\frac{2TP}{2TP+FN+FP}$$

(3) $$Mcc=\frac{TP\times TN-FP\times FN}{\sqrt{\left(TP+FN)(TP+FP)(TN+FN)(TN+FP\right)}}$$

(4) $$Jaccard=\frac{TP}{TP+FN+FP}$$

### 4.3 Implementation details

All experiments were conducted on a system equipped with an NVIDIA GeForce RTX 4090 GPU with 24 GB of memory, and all models were implemented and optimized using Python under the PyTorch deep learning framework. During training, the networks were trained for 200 epochs using the Adam optimizer with an initial learning rate of 1×10^-^³ to ensure stable convergence. Moreover, a batch size of 16 was adopted to balance training stability and GPU memory utilization, and all input images were uniformly resized to a resolution of 256×256 pixels.

**Figure 7:**
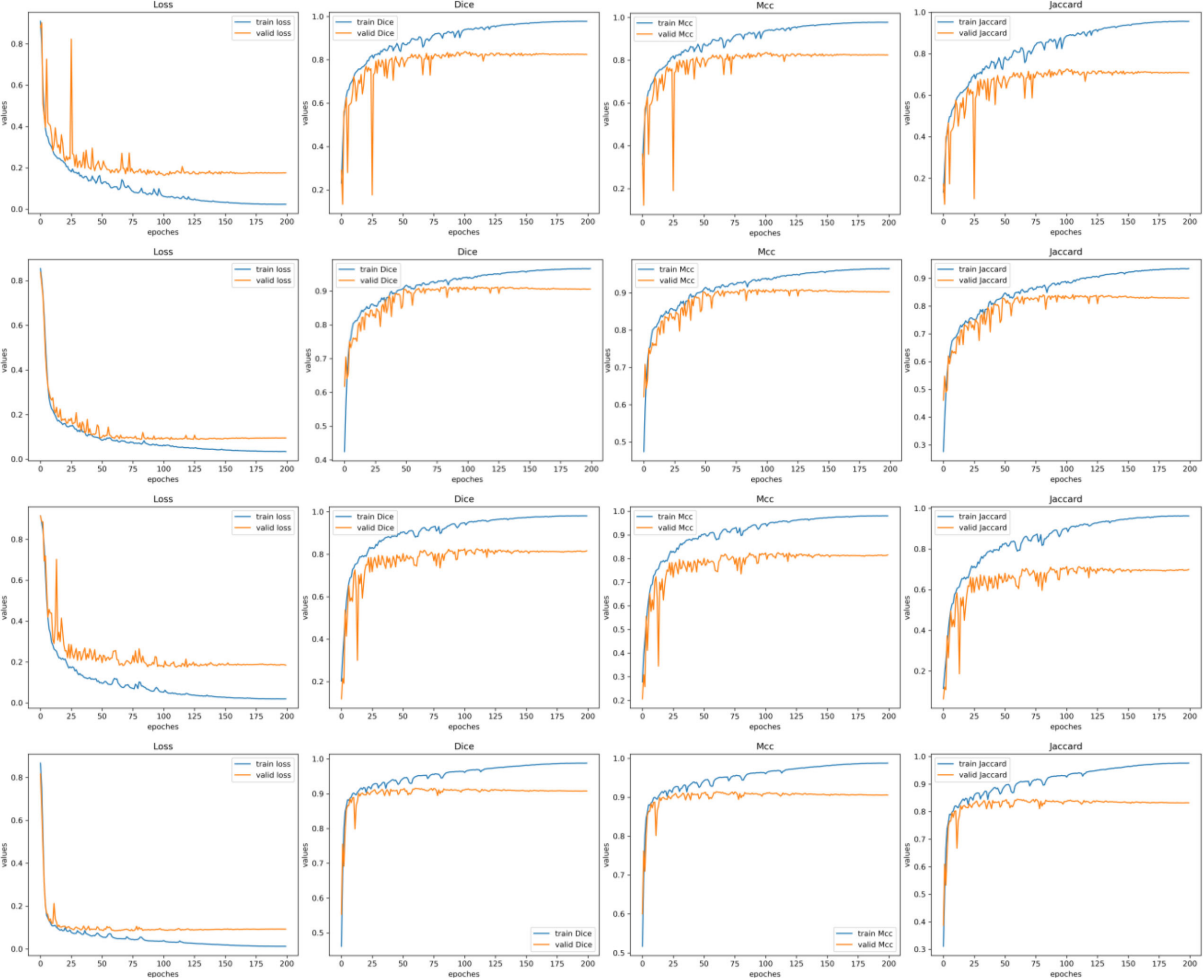
Training and validation curves of DGFI-Net across four brain tumor datasets. The rows from top to bottom represent the results on the BrainTumor1, BrainTumor2, BrainTumor3 and pituitary adenoma datasets.

Based on the training and validation curves shown in **Figure 7**, DGFI-Net demonstrates stable and efficient convergence across all four brain tumor datasets. For all metrics, including Loss, Dice, Mcc, and Jaccard, the training curves exhibit rapid improvement in the early epochs, followed by gradual convergence and stable performance in later stages, indicating effective optimization behavior. On the BrainTumor2 and pituitary adenoma datasets, the validation curves closely follow the training curves with relatively minor fluctuations, suggesting strong generalization capability and a well-aligned data distribution. In contrast, the BrainTumor1 and BrainTumor3 datasets exhibit more pronounced oscillations in the validation metrics and larger gaps between training and validation performance, particularly for Dice and Jaccard scores. This behavior can be attributed to increased sample complexity, greater inter-subject variability, and more ambiguous tumor boundaries in these datasets. Notably, despite these challenges, DGFI-Net consistently maintains stable validation performance without severe degradation, highlighting its robustness and adaptability.

### 4.4 Ablation study

As summarized in **Table 1**, the ablation analysis was performed on the BrainTumor1 dataset to systematically assess the contribution of each architectural component in DGFI-Net. Specifically, the baseline U-Net achieves Dice coefficient of 0.8010 and is used as the reference for comparison. By incorporating the ECRB, the Dice and Jaccard scores increase to 0.8171 and 0.6998, demonstrating that enhanced contextual modeling in the encoder effectively improves segmentation accuracy. The integration of the AGFRB also yields noticeable performance gains, achieving Dice and Jaccard values of 0.8097 and 0.7090, which validates the role of attention mechanisms in emphasizing tumor-relevant features. When ECRB and AGFRB are jointly employed together with the dual-branch guided feature interaction (DGFI) strategy, the model achieves further improvements, with the Dice score rising to 0.8289 and the Jaccard score to 0.7152, indicating strong complementarity between contextual refinement and attention-guided encoding. Finally, the full DGFI-Net equipped with the LRM delivers the best performance across all evaluation metrics, reaching Dice, Mcc, and Jaccard scores of 0.8402, 0.8387, and 0.7318, respectively. Overall, these results clearly demonstrate that each proposed module contributes positively to performance enhancement, and their synergistic integration is crucial for achieving robust and accurate brain tumor segmentation.

**Table 1:** Ablation study results on the BrainTumor1 dataset.

Method	Dice	Mcc	Jaccard
Baseline (U-Net)	0.8010	0.8040	0.6785
Baseline+ECRB	0.8171	0.8161	0.6998
Baseline+AGFRB	0.8097	0.8168	0.7090
Baseline+ECRB+AGFRB+DGFI	0.8289	0.8274	0.7152
Baseline+ECRB+AGFRB+DGFI+LRM (DGFI-Net)	0.8402	0.8387	0.7318

As shown in **Table 2**, introducing a bottleneck enhancement module consistently improves segmentation performance, highlighting the importance of strengthening high-level semantic representations. Among the compared methods, various attention-based modules achieve moderate improvements by reweighting feature responses. However, their gains are relatively limited since they mainly focus on channel-wise or spatial attention without fully exploiting joint feature interactions. In contrast, the proposed lightweight reinforcement module achieves the best performance across all metrics, with Dice, Mcc, and Jaccard scores of 0.8402, 0.8387, and 0.7318, respectively. This superior performance can be attributed to its ability to simultaneously model spatial and channel-wise features through the integration of depthwise convolution, pointwise convolution, and residual aggregation. Unlike conventional attention mechanisms that rely primarily on feature reweighting, LRM enhances feature representations in a more comprehensive manner while maintaining low computational complexity. These results demonstrate that LRM provides a more effective and efficient strategy for bottleneck feature enhancement, leading to improved segmentation accuracy and more precise boundary delineation.

**Table 2:** Ablation study of different bottleneck enhancement modules on the BrainTumor1 dataset.

Method	Dice	Mcc	Jaccard
Channel attention	0.8368	0.8351	0.7261
Convolutional block attention module	0.8234	0.8215	0.7078
Criss cross attention	0.8290	0.8280	0.7166
Efficient channel attention	0.8311	0.8303	0.7182
Large kernel attention	0.8287	0.8273	0.7140
Multi aspect global context attention	0.8310	0.8299	0.7188
Simple attention module	0.8330	0.8313	0.7206
Spatial attention	0.8366	0.8349	0.7259
Spatial large kernel attention	0.8295	0.8288	0.7176
Lightweight reinforcement module	0.8402	0.8387	0.7318

### 4.5 Comparison study

To rigorously evaluate the performance of DGFI-Net, extensive comparative experiments were conducted against several representative and state-of-the-art deep learning models. The selected methods include I2U-Net (Dai et al., 2024), TRFE-Net (Gong et al., 2023), DESENet (Tang et al., 2024a), TransUNet (Chen et al., 2021), TopFormer (Zhang et al., 2022), Swin-unetr (Hatamizadeh et al., 2022), CSC-UNet (Tang et al., 2024b), IMFF-Net (Liu et al., 2024), A-Net (Chen et al., 2023), PMFSNet (Zhong et al., 2025), and NLIE-UNet (Wan et al., 2025c). These comparison models cover a diverse range of architectural paradigms to ensure a comprehensive and fair evaluation. Specifically, I2U-Net, TRFE-Net, and DESENet are representative dual-branch architectures, which emphasize multi-path feature learning. TransUNet, TopFormer, and Swin-unetr are Transformer-based approaches that focus on capturing long-range dependencies and global contextual information. The remaining models, including CSC-UNet, IMFF-Net, A-Net, PMFSNet, and NLIE-UNet, are based on the conventional encoder-decoder framework, serving as strong baselines for medical image segmentation tasks. A detailed summary of these comparison methods, including their key characteristics, is provided in **Table 3**.

**Table 3:** Detailed description of comparison methods.

Method	Type	Description
I2U-Net (Dai et al., 2024)	Dual-branch	The dual-path U-Net framework integrates a multi-functional information interaction module designed to achieve comprehensive feature exchange across different pathways, hierarchical levels, and their cross-level interactions.
TRFE-Net (Gong et al., 2023)	Dual-branch	A dual-task learning framework integrates a prior-based feature enhancement module to fuse prior information with learned feature representations.
DESENet (Tang et al., 2024a)	Dual-branch	A parallel branch is constructed within the encoder-decoder framework, consisting of a semantic feature extraction module and a spatial feature extraction module.
TransUNet (Chen et al., 2021)	Transformer	Within the U-Net architecture, the transformer encodes tokenized image patches derived from CNN feature maps into input sequences.
TopFormer (Zhang et al., 2022)	Transformer	The TopFormer takes tokens at multiple scales as input to generate scale-aware semantic features, which are subsequently integrated into the corresponding tokens to enhance the feature representation.
Swin-unetr (Hatamizadeh et al., 2022)	Transformer	Swin-unetr employs a hierarchical swin transformer as the encoder, where self-attention is computed using a shifted window mechanism.
CSC-Unet (Tang et al., 2024b)	Encoder-decoder	A convolutional sparse coding strategy is integrated into the U-Net framework to improve feature representation and promote more efficient encoding of salient structures.
IMFF-Net (Liu et al., 2024)	Encoder-decoder	An attention pooling feature fusion module is designed in the U-shaped network for decoding, which consists of max pooling, average pooling, and a squeeze-and-excitation module.
A-Net (Chen et al., 2023)	Encoder-decoder	The A-shaped architecture employs a series of lightweight feature extraction modules to improve feature learning efficiency while maintaining low computational complexity.
PMFSNet (Zhong et al., 2025)	Encoder-decoder	The hierarchical design of the U-Net architecture is enhanced while reducing the computational burden of the self-attention mechanism.
NLIE-UNet (Wan et al., 2025c)	Encoder-decoder	The U-Net architecture employs a recurrent dynamic convolution block and a hierarchical interwoven fusion module for cross-level feature integration.

As shown in **Table 4**, the quantitative comparison results on the BrainTumor1 dataset are presented to evaluate the segmentation performance of different methods. DGFI-Net achieves the best performance across all evaluation metrics, with Dice, Mcc, and Jaccard scores of 0.8402, 0.8387, and 0.7318, demonstrating its effectiveness for brain tumor segmentation. Among the compared methods, the dual-branch models, including I2U-Net, TRFE-Net, and DESENet, generally exhibit competitive performance due to their ability to exploit complementary feature representations from multiple pathways, with TRFE-Net achieving relatively strong results (Dice of 0.8352, Mcc of 0.8348, Jaccard of 0.7253). Transformer-based methods, such as TransUNet, TopFormer, and Swin-unetr, show comparatively lower performance, which may be attributed to their reliance on token-based representations and the challenges in capturing fine-grained details in medical images. In contrast, the encoder-decoder-based approaches, including CSC-UNet, IMFF-Net, A-Net, PMFSNet, and NLIE-UNet, demonstrate relatively stable performance, with Dice scores mostly ranging from 0.80 to 0.83, benefiting from effective hierarchical feature extraction but still limited in modeling complex feature interactions. Overall, DGFI-Net outperforms all competing methods by effectively integrating cross-branch feature interaction and enhancing both contextual representation and fine-grained detail modeling, leading to improved segmentation accuracy and boundary delineation.

**Table 4:** Comparative study on the BrainTumor1 dataset.

Method	Dice	Mcc	Jaccard
I2U-Net (Dai et al., 2024)	0.8252	0.8246	0.7106
TRFE-Net (Gong et al., 2023)	0.8352	0.8348	0.7253
DESENet (Tang et al., 2024a)	0.8094	0.8086	0.6875
TransUNet (Chen et al., 2021)	0.7448	0.7498	0.6077
TopFormer (Zhang et al., 2022)	0.7238	0.7238	0.5766
Swin-unetr (Hatamizadeh et al., 2022)	0.5782	0.5780	0.4193
CSC-Unet (Tang et al., 2024b)	0.8271	0.8257	0.7105
IMFF-Net (Liu et al., 2024)	0.8330	0.8327	0.7218
A-Net (Chen et al., 2023)	0.8165	0.8167	0.7004
PMFSNet (Zhong et al., 2025)	0.8072	0.8065	0.6838
NLIE-UNet (Wan et al., 2025c)	0.8214	0.8200	0.7045
DGFI-Net	0.8402	0.8387	0.7318

**Figure 8:**
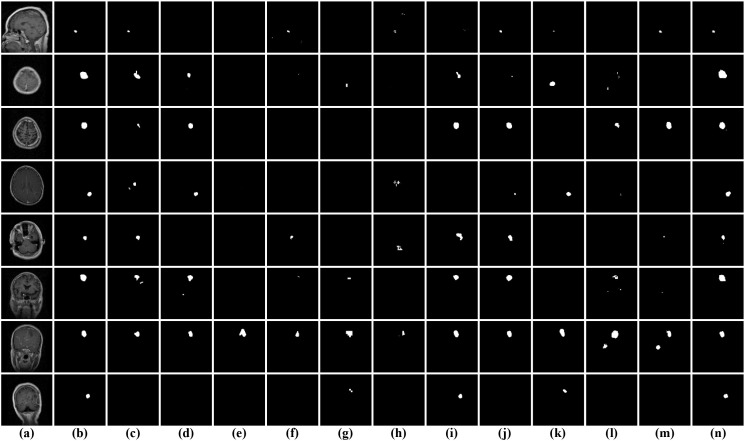
Visual comparison of segmentation results on the BrainTumor1 dataset. **(a)** input images; **(b)** ground truth; **(c)** I2U-Net; **(d)** TRFE-Net; **(e)** DESENet; **(f)** TransUNet; **(g)** TopFormer; **(h)** Swin-unetr; **(i)** CSC-Unet; **(j)** IMFF-Net; **(k)** A-Net; **(l)** PMFSNet; **(m)** NLIE-UNet; **(n)** DGFI-Net.

As illustrated in **Figure 8** qualitative comparisons of segmentation results on the BrainTumor1 dataset are presented for different methods. It can be observed that significant variations exist among the models in terms of lesion localization, boundary delineation, and noise suppression. Among the dual-branch models, I2U-Net and TRFE-Net are able to roughly localize tumor regions in most cases, but their predictions tend to be incomplete, with missing regions and insufficient coverage of the lesion areas. In addition, both methods show limitations in preserving fine structural details, particularly for small or low-contrast tumors, where fragmented or weak responses can be observed. Compared with these methods, DESENet exhibits more unstable performance, as it frequently produces inaccurate or scattered predictions, and in several cases fails to correctly identify the tumor regions. Transformer-based approaches show relatively weaker performance in capturing fine-grained structures. These methods often generate coarse segmentation maps, with noticeable false negatives and discontinuities, especially for small or low-intensity tumor regions. This suggests that although Transformer models are effective in modeling global context, they may struggle to preserve local details in medical image segmentation tasks. Among the encoder-decoder-based methods, the segmentation results are generally more stable, with most tumor regions being correctly identified. However, in some cases, slight over-segmentation or under-segmentation can be observed. Moreover, for small or low-contrast tumor regions, these models may fail to capture fine structural details, resulting in incomplete or less accurate predictions. In contrast, DGFI-Net generates more accurate and consistent segmentation results across different samples. It not only precisely localizes tumor regions but also preserves fine structural details and produces smoother yet well-defined boundaries. Moreover, DGFI-Net effectively suppresses background noise and avoids fragmented predictions, particularly in challenging scenarios with small or low-contrast lesions. These observations demonstrate that the proposed DGFI-Net achieves superior visual performance by effectively integrating cross-branch feature interaction and enhancing both global contextual representation and local detail modeling.

**Figure 9:**
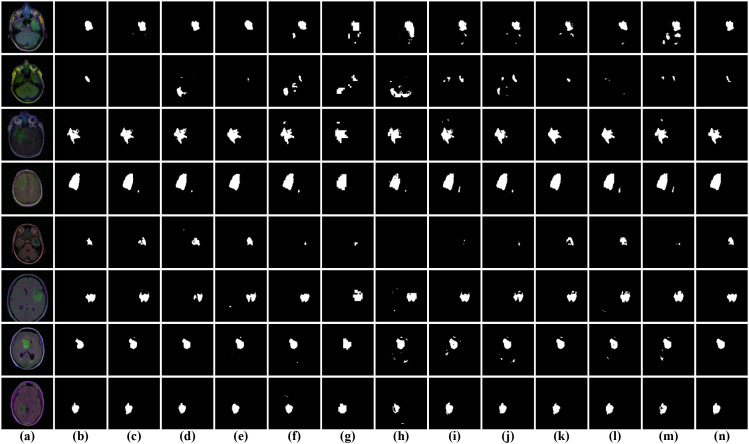
Visual comparison of segmentation results on the BrainTumor2 dataset. **(a)** input images; **(b)** ground truth; **(c)** I2U-Net; **(d)** TRFE-Net; **(e)** DESENet; **(f)** TransUNet; **(g)** TopFormer; **(h)** Swin-unetr; **(i)** CSC-Unet; **(j)** IMFF-Net; **(k)** A-Net; **(l)** PMFSNet; **(m)** NLIE-UNet; **(n)** DGFI-Net.

**Table 5** reports the quantitative comparison results on the BrainTumor2 dataset. Compared with the results on BrainTumor1, all methods achieve higher scores across Dice, Mcc, and Jaccard, indicating that BrainTumor2 presents relatively clearer structures and is less challenging for segmentation. For the dual-branch models, including I2U-Net, TRFE-Net, and DESENet, competitive performance is observed, with Dice values generally above 0.88, reflecting the effectiveness of multi-path feature learning. Transformer-based methods still show comparatively lower performance, suggesting that their ability to capture fine-grained details remains limited despite strong global modeling capability. In contrast, encoder-decoder-based methods exhibit relatively stable results, with most Dice scores exceeding 0.88, demonstrating their reliability in hierarchical feature extraction. Among all approaches, DGFI-Net achieves the best overall performance, with Dice, Mcc, and Jaccard scores of 0.9011, 0.8984, and 0.8213, indicating its effectiveness in balancing the modeling of the global background and local details. The visual comparisons in **Figure 9** further confirm these observations. Since most methods are able to correctly localize tumor regions on this dataset, the overall differences are less pronounced than those observed on BrainTumor1. However, distinctions can still be observed in boundary refinement and detail preservation. DGFI-Net produces more continuous and accurate boundaries, while better preserving small structures and reducing isolated artifacts. These results indicate that DGFI-Net maintains more consistent and refined segmentation quality, which aligns with its quantitative advantages.

**Table 5:** Comparative study on the BrainTumor2 dataset.

Method	Dice	Mcc	Jaccard
I2U-Net (Dai et al., 2024)	0.8969	0.8943	0.8144
TRFE-Net (Gong et al., 2023)	0.8936	0.8911	0.8094
DESENet (Tang et al., 2024a)	0.8881	0.8854	0.8004
TransUNet (Chen et al., 2021)	0.8806	0.8780	0.7886
TopFormer (Zhang et al., 2022)	0.8220	0.8181	0.6992
Swin-unetr (Hatamizadeh et al., 2022)	0.7587	0.7621	0.6337
CSC-Unet (Tang et al., 2024b)	0.8981	0.8957	0.8165
IMFF-Net (Liu et al., 2024)	0.8992	0.8966	0.8185
A-Net (Chen et al., 2023)	0.8925	0.8900	0.8071
PMFSNet (Zhong et al., 2025)	0.8823	0.8801	0.7920
NLIE-UNet (Wan et al., 2025c)	0.8981	0.8956	0.8167
DGFI-Net	0.9011	0.8984	0.8213

**Figure 10:**
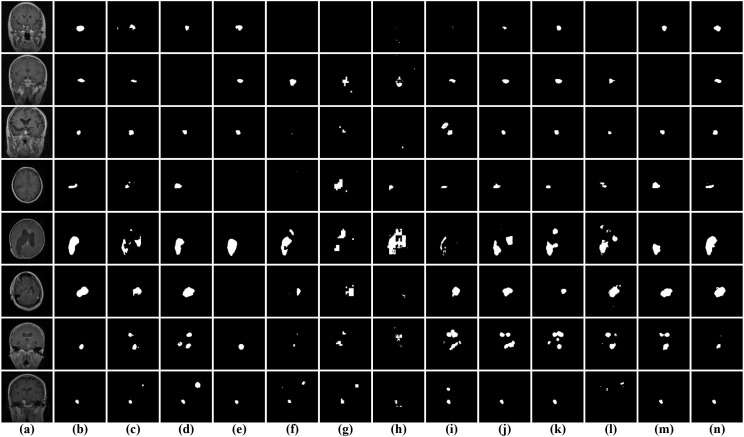
Visual comparison of segmentation results on the BrainTumor3 dataset. **(a)** input images; **(b)** ground truth; **(c)** I2U-Net; **(d)** TRFE-Net; **(e)** DESENet; **(f)** TransUNet; **(g)** TopFormer; **(h)** Swin-unetr; **(i)** CSC-Unet; **(j)** IMFF-Net; **(k)** A-Net; **(l)** PMFSNet; **(m)** NLIE-UNet; **(n)** DGFI-Net.

**Table 6** summarizes the quantitative results on the BrainTumor3 dataset. Compared with BrainTumor1 and BrainTumor2, all methods show noticeably lower performance across Dice, Mcc, and Jaccard, indicating that BrainTumor3 is more challenging due to increased lesion variability and more complex imaging characteristics. Among the compared methods, IMFF-Net achieves relatively strong performance with scores of 0.7851, 0.7848, and 0.6737, while NLIE-UNet obtains comparable results of 0.7835, 0.7834, and 0.6698. Dual-branch models such as I2U-Net and TRFE-Net also demonstrate stable performance, with Dice values around 0.78. In contrast, Transformer-based methods, including TransUNet, TopFormer, and Swin-unetr, exhibit lower performance, particularly in terms of region overlap consistency. Encoder-decoder-based methods show moderate results overall, with PMFSNet achieving the lowest scores of 0.7429, 0.7436, and 0.6169. Despite the overall performance degradation on this dataset, DGFI-Net still achieves the best results across all metrics, with Dice, Mcc, and Jaccard values of 0.8040, 0.8021, and 0.6899, respectively, maintaining a clear advantage under more challenging conditions. The qualitative comparisons in **Figure 10** further highlight the increased difficulty of BrainTumor3. Compared with the clearer segmentation results observed on BrainTumor2, many methods exhibit more pronounced over-segmentation, with scattered false-positive regions appearing around the tumor or in normal tissues. In addition, fragmented predictions and irregular boundaries are frequently observed, especially for small or low-contrast lesions. Although several models can still roughly localize tumor regions, their outputs are often affected by noisy artifacts or exaggerated segmentation regions. In contrast, DGFI-Net demonstrates better suppression of false positives and produces more compact and coherent segmentation results, with improved boundary consistency. These findings indicate that BrainTumor3 poses greater challenges for accurate segmentation, while DGFI-Net maintains superior robustness and stability compared with existing methods.

**Table 6:** Comparative study on the BrainTumor3 dataset.

Method	Dice	Mcc	Jaccard
I2U-Net (Dai et al., 2024)	0.7840	0.7865	0.6701
TRFE-Net (Gong et al., 2023)	0.7884	0.7878	0.6732
DESENet (Tang et al., 2024a)	0.7607	0.7635	0.6438
TransUNet (Chen et al., 2021)	0.6778	0.6900	0.5419
TopFormer (Zhang et al., 2022)	0.6847	0.6839	0.5378
Swin-unetr (Hatamizadeh et al., 2022)	0.5256	0.5274	0.3766
CSC-Unet (Tang et al., 2024b)	0.7522	0.7546	0.6337
IMFF-Net (Liu et al., 2024)	0.7851	0.7848	0.6737
A-Net (Chen et al., 2023)	0.7704	0.7684	0.6448
PMFSNet (Zhong et al., 2025)	0.7429	0.7436	0.6169
NLIE-UNet (Wan et al., 2025c)	0.7835	0.7834	0.6698
DGFI-Net	0.8040	0.8021	0.6899

**Figure 11:**
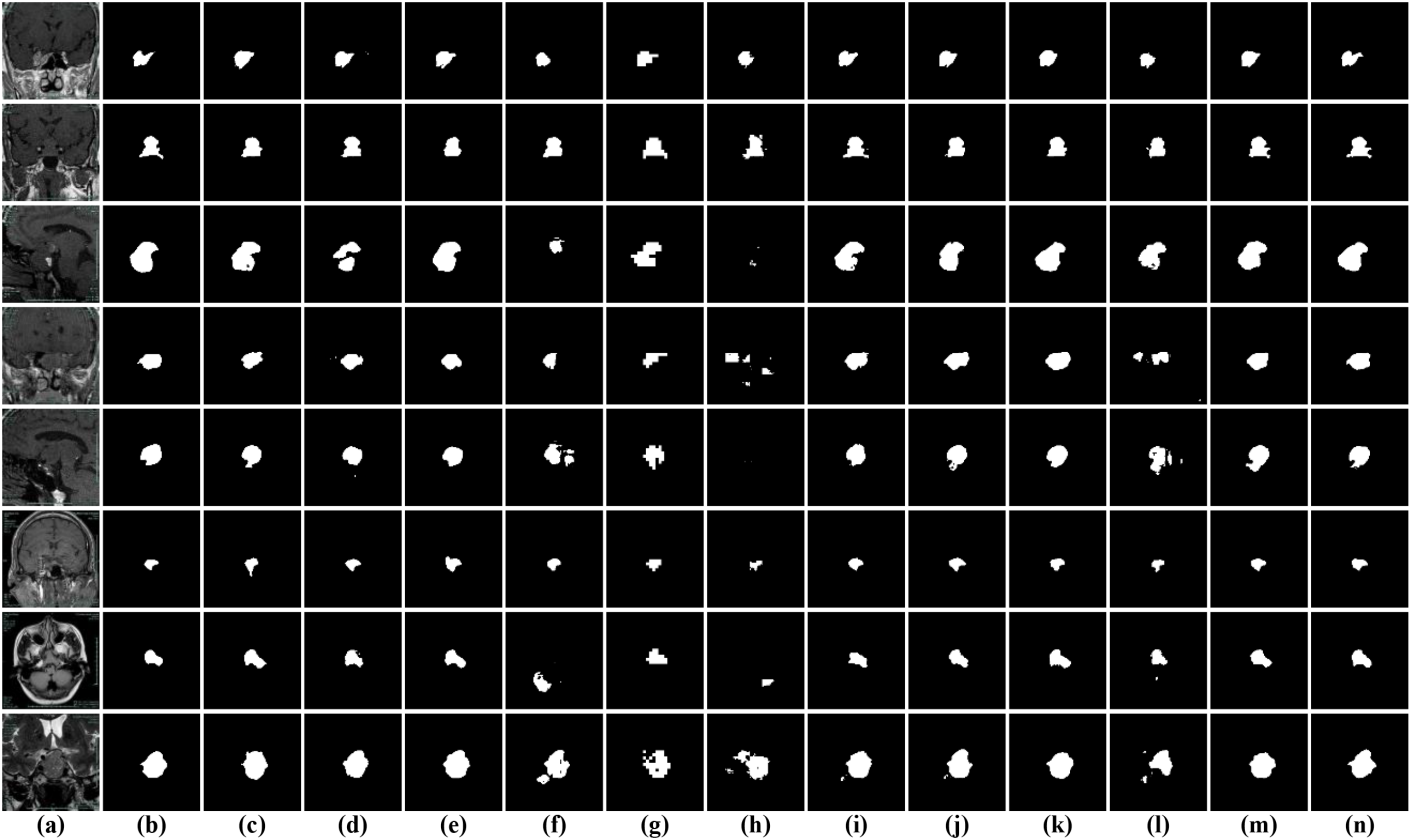
Visual comparison of segmentation results on the pituitary adenoma dataset. **(a)** input images; **(b)** ground truth; **(c)** I2U-Net; **(d)** TRFE-Net; **(e)** DESENet; **(f)** TransUNet; **(g)** TopFormer; **(h)** Swin-unetr; **(i)** CSC-Unet; **(j)** IMFF-Net; **(k)** A-Net; **(l)** PMFSNet; **(m)** NLIE-UNet; **(n)** DGFI-Net.

**Table 7** presents the quantitative evaluation results on the pituitary adenoma dataset. Compared with the BrainTumor1, BrainTumor2, and BrainTumor3 datasets, all methods achieve noticeably higher metric values on this dataset, indicating that pituitary adenoma segmentation is relatively less challenging in terms of lesion contrast and structural clarity. Most competing approaches obtain Dice scores above 0.90, demonstrating generally strong segmentation capability on this dataset. In particular, IMFF-Net, A-Net, NLIE-UNet, and CSC-UNet all exhibit stable and competitive performance, with Dice values exceeding 0.906. Among all methods, DGFI-Net achieves the best overall results, with Dice, Mcc, and Jaccard scores of 0.9117, 0.9100, and 0.8388, slightly outperforming A-Net and IMFF-Net. Although the performance differences among the top-performing models are relatively small, DGFI-Net consistently ranks first across all evaluation metrics, indicating its ability to achieve more balanced and reliable segmentation performance. The qualitative comparisons in **Figure 11** further support these findings. Compared with the brain tumor datasets, most methods on the pituitary adenoma dataset are able to generate relatively complete and well-defined tumor regions, with fewer false-positive predictions. However, subtle differences can still be observed in boundary accuracy and fine detail representation. Some methods produce slightly irregular contours or minor deviations along complex boundaries, particularly in regions with subtle intensity transitions. In contrast, DGFI-Net produces segmentation results that are more consistent with the ground truth, exhibiting smoother boundaries and more precise delineation, especially in edge and small-structure regions. Overall, both quantitative and qualitative results demonstrate that although the task is relatively less challenging and most methods perform well, DGFI-Net still achieves the most accurate and refined segmentation results, further validating its robustness and effectiveness.

**Table 7:** Comparative study on the pituitary adenoma dataset.

Method	Dice	Mcc	Jaccard
I2U-Net (Dai et al., 2024)	0.9046	0.9027	0.8274
TRFE-Net (Gong et al., 2023)	0.9074	0.9056	0.8322
DESENet (Tang et al., 2024a)	0.9003	0.8983	0.8203
TransUNet (Chen et al., 2021)	0.8630	0.8619	0.7640
TopFormer (Zhang et al., 2022)	0.8478	0.8448	0.7374
Swin-unetr (Hatamizadeh et al., 2022)	0.8103	0.8079	0.6870
CSC-Unet (Tang et al., 2024b)	0.9065	0.9047	0.8300
IMFF-Net (Liu et al., 2024)	0.9105	0.9088	0.8370
A-Net (Chen et al., 2023)	0.9111	0.9093	0.8379
PMFSNet (Zhong et al., 2025)	0.8959	0.8940	0.8132
NLIE-UNet (Wan et al., 2025c)	0.9091	0.9072	0.8345
DGFI-Net	0.9117	0.9100	0.8388

### 4.6 Computational efficiency

**Table 8** presents the computational efficiency of different methods on the BrainTumor1 dataset in terms of GFLOPs, number of parameters, and inference speed (FPS). It can be observed that there are significant differences among the compared models in terms of computational cost and efficiency. Among the dual-branch models, I2U-Net and DESENet exhibit relatively low computational complexity, with GFLOPs of 3.56 and 3.17, respectively. DESENet is particularly lightweight, with only 1.16M parameters, while I2U-Net maintains moderate model capacity. In contrast, TRFE-Net introduces substantially higher computational overhead, with 92.39 GFLOPs and 43.24M parameters, making it less efficient despite its competitive segmentation performance. For Transformer-based methods, TransUNet and Swin-unetr require relatively high computational resources due to the self-attention mechanism, with TransUNet having the largest number of parameters (66.82M). Although TopFormer is designed as a lightweight Transformer model with only 0.42 GFLOPs, its parameter count (5.02M) and inference speed are still less competitive compared with lightweight convolutional approaches. Among encoder-decoder-based models, several methods demonstrate strong efficiency advantages. In particular, A-Net and PMFSNet achieve extremely low computational complexity, with GFLOPs below 1 and parameters under 0.5M, making them highly lightweight. CSC-UNet and IMFF-Net achieve high inference speeds, with FPS values of 243.91 and 258.13, respectively, indicating their efficiency in practical deployment scenarios. However, these models may sacrifice certain representational capacity or feature interaction ability for efficiency. In comparison, DGFI-Net achieves a favorable balance between computational cost and segmentation performance. With 9.01 GFLOPs and 6.81M parameters, it maintains a moderate model complexity while achieving a high inference speed of 230.41 FPS. Compared with lightweight models, DGFI-Net provides significantly improved segmentation accuracy, and compared with heavy models such as TRFE-Net and TransUNet, it achieves substantially lower computational overhead. These results demonstrate that DGFI-Net effectively balances efficiency and performance, making it suitable for real-world medical image segmentation tasks.

**Table 8:** Computational efficiency on the BrainTumor1 dataset.

Method	GFLOPs	Params(M)	FPS
I2U-Net (Dai et al., 2024)	3.56	6.76	43.03
TRFE-Net (Gong et al., 2023)	92.39	43.24	198.70
DESENet (Tang et al., 2024a)	3.17	1.16	133.56
TransUNet (Chen et al., 2021)	32.55	66.82	124.63
TopFormer (Zhang et al., 2022)	0.42	5.02	122.98
Swin-unetr (Hatamizadeh et al., 2022)	11.36	41.34	133.84
CSC-Unet (Tang et al., 2024b)	11.46	0.52	243.91
IMFF-Net (Liu et al., 2024)	71.96	34.68	258.13
A-Net (Chen et al., 2023)	0.60	0.39	184.24
PMFSNet (Zhong et al., 2025)	0.59	0.33	144.05
NLIE-UNet (Wan et al., 2025c)	7.17	4.05	114.11
DGFI-Net	9.01	6.81	230.41

### 4.7 Selection of loss function

To evaluate the impact of different loss functions on the performance of DGFI-Net, comparative experiments were conducted on the BrainTumor1 dataset, as shown in **Table 9**. Five commonly used loss functions, including Dice, binary cross-entropy (BCD), Hausdorff, IoU, and Tversky losses, were tested under identical training settings. Among them, Dice loss achieves the best performance, with Dice, Mcc, and Jaccard scores of 0.8402, 0.8387, and 0.7318, respectively. In comparison, BCD yields lower results, with Dice decreasing to 0.8034 and Jaccard to 0.6809, suggesting that pixel-wise supervision alone is less effective for optimizing region-level overlap. Hausdorff loss further reduces performance, while IoU and Tversky losses show substantially inferior results, with Dice values below 0.50, indicating unstable convergence and limited optimization capability in this task. These findings demonstrate that Dice loss provides the most suitable optimization objective for DGFI-Net, as it directly maximizes region overlap and better addresses the foreground-background imbalance inherent in brain tumor segmentation.

**Table 9:** Selection of loss function on the BrainTumor1 dataset.

Loss function	Dice	Mcc	Jaccard
Dice	0.8402	0.8387	0.7318
BCD	0.8034	0.8010	0.6809
Hausdorff	0.6258	0.6520	0.4826
IoU	0.3825	0.4667	0.2381
Tversky	0.4177	0.4936	0.2660

## 5 Conclusion

In this paper, we presented DGFI-Net, a guided dual-branch feature interaction network for brain tumor segmentation. By introducing an auxiliary branch to progressively guide the representation learning of the main branch, the proposed framework enhances feature complementarity and improves structural consistency in tumor delineation. The designed feature refinement and interaction strategies enable more effective integration of contextual information and salient region emphasis, leading to more accurate and stable segmentation results. Extensive evaluations on three public brain tumor benchmark datasets and an additional pituitary adenoma dataset demonstrate that DGFI-Net achieves superior performance compared with existing state-of-the-art methods. The proposed model shows strong robustness across datasets with varying difficulty levels and maintains competitive computational efficiency. Furthermore, ablation experiments confirm that each component contributes positively to the overall segmentation performance.

Overall, the proposed guided dual-branch interaction paradigm provides an effective solution for handling complex tumor morphology and ambiguous boundaries, highlighting its potential for reliable and automated medical image analysis. From a clinical perspective, the accurate and consistent segmentation results produced by DGFI-Net may assist clinicians in tumor localization, treatment planning, and disease monitoring. In addition, its favorable trade-off between computational efficiency and segmentation accuracy makes it suitable for integration into computer-aided diagnosis systems and clinical workflows. Nevertheless, further validation on larger multi-center datasets is required to fully assess its generalization capability.

## Data Availability

The raw data supporting the conclusions of this article will be made available by the authors, without undue reservation.
